# Information-theoretic gradient flows in mouse visual cortex

**DOI:** 10.3389/fninf.2025.1700481

**Published:** 2025-10-30

**Authors:** Erik D. Fagerholm, Hirokazu Tanaka, Milan Brázdil

**Affiliations:** 1 First Department of Neurology, St. Anne’s University Hospital and Faculty of Medicine, Masaryk University, Brno, Czechia; 2 Faculty of Information Technology, Tokyo City University, Tokyo, Japan

**Keywords:** information geometry, gradient flows, neural connectivity, entropy, expectation, two photon, calcium imaging

## Abstract

**Introduction:**

Neural activity can be described in terms of probability distributions that are continuously evolving in time. Characterizing how these distributions are reshaped as they pass between cortical regions is key to understanding how information is organized in the brain.

**Methods:**

We developed a mathematical framework that represents these transformations as information-theoretic gradient flows — dynamical trajectories that follow the steepest ascent of entropy and expectation. The relative strengths of these two functionals provide interpretable measures of how neural probability distributions change as they propagate within neural systems. Following construct validation *in silico*, we applied the framework to publicly available continuous ΔF/F two-photon calcium recordings from the mouse visual cortex.

**Results:**

The analysis revealed consistent bi-directional transformations between the rostrolateral area and the primary visual cortex across all five mice. These findings demonstrate that the relative contributions of entropy and expectation can be disambiguated and used to describe information flow within cortical networks.

**Discussion:**

We introduce a framework for decomposing neural signal transformations into interpretable information-theoretic components. Beyond the mouse visual cortex, the method can be applied to diverse neuroimaging modalities and scales, thereby providing a generalizable approach for quantifying how information geometry shapes cortical communication.

## Introduction

The electrical activity in the brain reflects a combination of hidden internal states which, although not directly observable, can be inferred via the signals picked up by neuroimaging devices (Fiser et al., 2010; Friston, 2005; Ma et al., 2006). One way to describe these signals is in terms of probability distributions evolving in time. As conditions change in the brain, the probability distributions shift accordingly, reflecting an ongoing reorganization of internal representations. Understanding the processes by which probability distributions transform as they pass among brain regions remains a central challenge in computational neuroscience.

Changes in neural activity can be analyzed by studying how specific functionals act on probability distributions. Two key examples of such functionals are entropy (Fagerholm et al., 2023; Keshmiri, 2020; Luczak, 2024) and expectation (Gerstner and Kistler, 2002; Helias et al., 2014; Lánskı and Sacerdote, 2001), where the former widens the variance and the latter shifts the mean of a given probability distribution. Each functional is associated, via its gradient, with a specific flow across the space of probability densities. This geometric (Nielsen, 2022) perspective allows for a decomposition of transformations into interpretable information-theoretic components.

We describe how neural activity distributions change when viewed at different observational scales, and formalize these changes using information-theoretic geometry. The adaptation of neural distributions, central to predictive coding (Clark, 2013; Rao and Ballard, 1999) and efficient representation (Barlow, 1961; Simoncelli and Olshausen, 2001; Wei and Stocker, 2015), corresponds to systematic transformations in probability space. By expressing these transformations as gradient flows, we provide a mathematical description of how distributions evolve under the competing influences of variability (entropy) and stability (expectation). We show that in the specific case of a centered symmetric distribution, entropy and expectation form orthogonal components, and are thus able to be added as basis flows.

Previous work on neural signal transmissions has been largely focused on statistical dependencies between observed activation patterns (Bastos and Schoffelen, 2016; Friston, 2011). For instance, metrics such as mutual information (Borst and Theunissen, 1999; Panzeri et al., 2017) and Granger causality (Seth et al., 2015) quantify how strongly activity in one region predicts activity in another. However, these metrics do not capture how the full probability distributions transform across regions. This is precisely the missing component that our methodology addresses.

While the present study applies the framework to continuous ΔF/F calcium signals, the formulation itself is modality-agnostic. Because it operates on empirical probability densities, rather than on “raw” measurements, the same principles can be applied to spiking activity. This is achieved by constructing firing-rate distributions or by using smooth approximations of Poisson processes — a standard approach in population coding models.

We validate this framework *in silico* and then extract dominant flows linking regions within the murine visual cortex, captured using two-photon imaging. The visual cortex in mice is particularly well-suited to our study, given that adjacent areas therein exhibit coordinated patterns of activity (Felleman and Van Essen, 1991; Harris et al., 2019) across functionally specialized regions (Andermann and Moore, 2006; Glickfeld et al., 2013; Marshel et al., 2011). Beyond this specific application, our approach introduces a generalizable method for analysing *any* scenario in which distributions are transformed — not just among cortical regions, but also between measurement devices, or across spatiotemporal scales.

## Materials and methods

Here we formalize how probability distributions transform when the observation scale changes. This formulation reveals two flows — one linked to entropy and the other to expectation. In the case of a centered symmetric distribution these two flows form orthogonal bases for information-theoretic transformations.

We begin with the following definitions:

*x* ∈ ℝ^*n*^: the state of the system, represented by an *n*-dimensional variable.
λ_*S*_ ∈ ℝ^+^: a positive-valued parameter that controls the scale of observation.
*q* (*x*; λ_*S*_): a probability density function over *x*, conditioned on the observation scale λ_*S*_, which remains normalized for all scales:

$$\int qdx=1\,\forall \lambda_{S}.$$ (1)

We define the space of all valid (smooth, positive, normalized) probability distributions as the *information space* 𝒫:


$$𝒫=\left\{q\in C^{1}({\mathbb{R}}^{n})|q(x)>0,\int q(x)dx=1\right\},$$ (2)

which yields a nonlinear manifold of valid distributions within the space of all possible functions.

Power law generators: Due to the ubiquity of power laws in the analysis of neural systems (Fiser et al., 2010), we investigate how the probability distribution *q* (*x*; λ_*S*_) changes to a new distribution $\tilde{q}\,\left(x;\lambda_{S}\right)$ via:


$$\tilde{q}≜\frac{q^{\lambda_{S}}}{\int q^{\lambda_{S}}\,dx},$$ (3)

where the partition function in the denominator ensures correct normalization of the new distribution $\tilde{q}$ for all values of λ_*S*_.

We next analyze the form of Equation 3 for very small changes in scale. Specifically, we seek the associated generator (Amari and Nagaoka, 2000) — i.e., the infinitesimal power law transformation associated with an increase in λ_*S*_. As motivated by Noether’s theorem (Noether, 1983) and Lie theory (Cohn, 1957), the derivation of a generator creates a powerful tool that allows for the recovery of arbitrary transformations.

To see how this applies to our particular case, we begin by defining the scale parameter λ_*S*_ in terms of an arbitrarily small constant ε:


$$\lambda_{S}=1+\varepsilon ,$$ (4)

thereby allowing for *any* scale parameter λ_*S*_ to be defined by the iterated application of ε.

Applying Equation 4 to Equation 3, we obtain:


$$\tilde{q}≜\frac{q^{1+\varepsilon }}{\int q^{1+\varepsilon }\,dx}.$$ (5)

Next, using the fact that *e^x^* ≈ 1 + *x* for small *x*, we expand *q*^1 + ε^ to first order in ε and use the identities: *q*^1 + ε^ = *qq*^ε^, and *qq*^ε^ = *qe*^ε log ⁡ *q*^, to linearize the effect of the power law transform:


$$q^{1+\varepsilon }\approx \left(1+\varepsilon \,\log q\right)\,q,$$ (6)

which evaluates Equation 3 near λ_*S*_ = 1.

To ensure that the transformed density remains normalized, we divide Equation 6 by its associated partition function:


$$\tilde{q}≜\frac{1\,+\,\varepsilon \,l\,o\,g\,q}{\int qdx+\varepsilon \,\int q\,l\,o\,g\,q\,dx}\,q.$$ (7)

Substituting the normalization condition from Equation 1 into the denominator, and using the definition of the mean:


⟨l⁢o⁢g⁢q⟩=∫q⁢l⁢o⁢g⁢q⁢dx. (8)

Equation 7 simplifies to:


$$\tilde{q}≜\frac{1+\varepsilon \,l\,o\,g\,q}{1+\varepsilon \,\left\langle l\,o\,g\,q\right\rangle }\,q.$$ (9)

Finally, we use the fact that $\frac{1+x}{1+y}\approx 1+x-y$ for small *x* and *y* to linearize Equation 9, thereby yielding the power law generator:


$$\tilde{q}≜\left[1+\varepsilon \,\left(l\,o\,g\,q-\left\langle l\,o\,g\,q\right\rangle \right)\right]\,q,$$ (10)

which can equivalently be expressed as the following differential equation:


$$\frac{\partial l\,o\,g\,q}{\partial \lambda_{S}}=l\,o\,g\,q-\left\langle l\,o\,g\,q\right\rangle .$$ (11)

Power laws and entropic flow: We now note that the generator derived in Equation 10 includes a term *qlogq*, which resembles the integrand of entropy *S* [*q*], hinting at a connection between power law transformations and entropy:


S=-∫q⁢ log qd⁢x. (12)

We investigate this connection by calculating in which direction entropy increases most rapidly, within the space of valid probability distributions 𝒫 in Equation 2. This direction is given by the functional gradient of the negative entropy in Equation 12:


$$\frac{\delta \,S}{\delta \,q}=1+l\,o\,g\,q.$$ (13)

Equation 13 has a mean given by:


$$\left\langle \frac{\delta \,S}{\delta \,q}\right\rangle =\int qdx+\int qlogqdx,$$ (14)

which, using Equations 1, 8, can be written as:


$$\left\langle \frac{\delta \,S}{\delta \,q}\right\rangle =1+\left\langle l\,o\,g\,q\right\rangle .$$ (15)

We define an entropic flow *v_S_* as the mean gradient in Equation 15 subtracted from the gradient in Equation 13. This has the effect of projecting the gradient onto the manifold 𝒫 of valid probability densities in Equation 2:


$$v_{S}=\frac{\delta \,S}{\delta \,q}-\left\langle \frac{\delta \,S}{\delta \,q}\right\rangle ,$$ (16)

which, using Equations 13, 15, reads:


$$v_{S}=l\,o\,g\,q-\left\langle l\,o\,g\,q\right\rangle ,$$ (17)

i.e., we discover exactly the same expression as in Equation 11, meaning that we can write:


$$\frac{\partial l\,o\,g\,q}{\partial \lambda_{S}}=v_{S}.$$ (18)

This reveals a relationship between entropic flow and power law transformations indexed by a scale parameter λ_*S*_.

Generalized flow: The form of Equation 18 can be generalized to arbitrary functionals ℱ [*q*], which define continuous trajectories through information space 𝒫 via associated flow parameters λ_ℱ_. The flow of ℱ [*q*] preserves the geometric structure of Equation 18, in terms of a projected gradient on the log density of *q*, while allowing for arbitrary functionals:


$$\frac{\partial l\,o\,g\,q}{\partial \lambda_{ℱ}}=v_{ℱ},v_{ℱ}=\frac{\delta \,ℱ}{\delta \,q}-\left\langle \frac{\delta \,ℱ}{\delta \,q}\right\rangle .$$ (19)

Here, the *logq* term is not an artefact of the entropic expression in Equation 18. Rather, *logq* persists in the generalized flow expression in Equation 19 because λ_ℱ_ parameterizes a flow of the form ∂⁡*q*/∂⁡λ_ℱ_ ∼ *q*, which maps to ∂⁡*logq*/∂⁡λ_ℱ_. Equation 19 therefore yields a class of projected gradient flows *v*_*F*_ which depend on the choice of functional ℱ.

Basis flows: Thus far we have established that:

1. Power law transformations are associated with entropic flow,
2. The power law/entropy link can be generalized to arbitrary functionals beyond entropy.

Given these two points, our next question is whether we can find a flow *v*_ℱ_ that is orthogonal to entropic flow *v_S_*, as this would allow for a decomposition into independent components. To find such an orthogonal flow, we require that the inner product between *v*_ℱ_ and *v_S_* equals zero:


$$\left\langle v_{S},v_{ℱ}\right\rangle =0,$$ (20)

where we can use Equations 17, 19 to write the covariance as:


$$\left\langle v_{S},v_{ℱ}\right\rangle =\int \left(l\,o\,g\,q-\left\langle l\,o\,g\,q\right\rangle \right)\,\left(\frac{\delta \,ℱ}{\delta \,q}-\left\langle \frac{\delta \,ℱ}{\delta \,q}\right\rangle \right)\,q\,\left(x\right)\,dx,$$ (21)

which is equivalent to the covariance between *logq* and δℱ/δ*q* under *q* (*x*):


$$\left\langle v_{S},v_{ℱ}\right\rangle =C\,o\,v_{q}\,\left(l\,o\,g\,q,\frac{\delta \,ℱ}{\delta \,q}\right).$$ (22)

The simplest class of ℱ is given by linear expectation:


ℱ⁢[q]=∫x⁢q(x)dx, (23)

with a functional derivative given by:


$$\frac{\delta \,ℱ}{\delta \,q}=x.$$ (24)

If we then assume a zero-mean Gaussian form for *q* (*x*), for which *logq* ∼ *x*^2^, Equation 22 becomes:


$$\left\langle v_{S},v_{ℱ}\right\rangle =C\,o\,v_{q}\,\left(x^{2},x\right),$$ (25)

which satisfies the orthogonality condition in Equation 20, which in turn shows that entropy and expectation define orthogonal flows in the specific case of a centered symmetric distribution.

We next look for the transformation associated with the expectation functional using Equation 19:


$$\frac{\partial l\,o\,g\,q}{\partial \lambda }=x-\left\langle x\right\rangle ,$$ (26)

which has a solution given by:


l⁢o⁢g⁢q(x;λ)=l⁢o⁢g⁢q(x;0)+λ⁢(x-⟨x⟩), (27)

and hence:


$$q\,\left(x;\lambda \right)=\frac{q\,\left(x;0\right)\,e^{\lambda x}}{\int q\,\left(x;0\right)\,e^{\lambda x}\,dx},$$ (28)

where the partition function in the denominator ensures correct normalization.

Therefore, just as entropic flow arises from power law transformations in Equation 18, the expectation flow corresponds to an exponential tilt in Equation 28. Intuitively, the entropic and expectation flows capture how variance and expectation change with observational scale, respectively. We summarize the links between these two information-theoretic functionals and their associated geometric transformations in Table 1.

**TABLE 1:** Summary properties for entropy and expectation flows.

	Entropy	Expectation
Functional	ℱ = − ∫ *qlogqdx*	ℱ = ∫ *xq* (*x*) *dx*
Flow	$\frac{\partial l\,o\,g\,q}{\partial \lambda }=l\,o\,g\,q-\left\langle l\,o\,g\,q\right\rangle$	$\frac{\partial l\,o\,g\,q}{\partial \lambda }=x-\left\langle x\right\rangle$
Transformation	$q\to \frac{q^{\lambda }}{\int q^{\lambda }\,dx}$	$q\to \frac{q_{0}\,e^{\lambda x}}{\int q_{0}\,e^{\lambda \,x}\,dx}$

Synthetic data: Having established entropy and expectation as orthogonal basis functionals, we use Equations 17, 26 to define a mixed entropic-expectation flow combining both components:


$$\frac{\partial l\,o\,g\,q}{\partial \lambda }=\alpha \,\left(l\,o\,g\,q-\left\langle l\,o\,g\,q\right\rangle \right)+\beta \,\left(x-\left\langle x\right\rangle \right),$$ (29)

where the coefficients α and β control the relative contributions of entropy and expectation, respectively.

To verify that the model parameters can be accurately recovered from data, we performed two *in silico* tests. The flow in Equation 29 was simulated using pre-specified α and β-values applied to samples drawn from: (1) a Gaussian process, and (2) a one-dimensional Langevin process with a time-varying oscillatory drift term. Recovery accuracy was assessed by comparing true versus fitted parameters and evaluating similarity between distributions using Wasserstein-2 distance, total variation, and L^2^ metrics.

**FIGURE 1:**
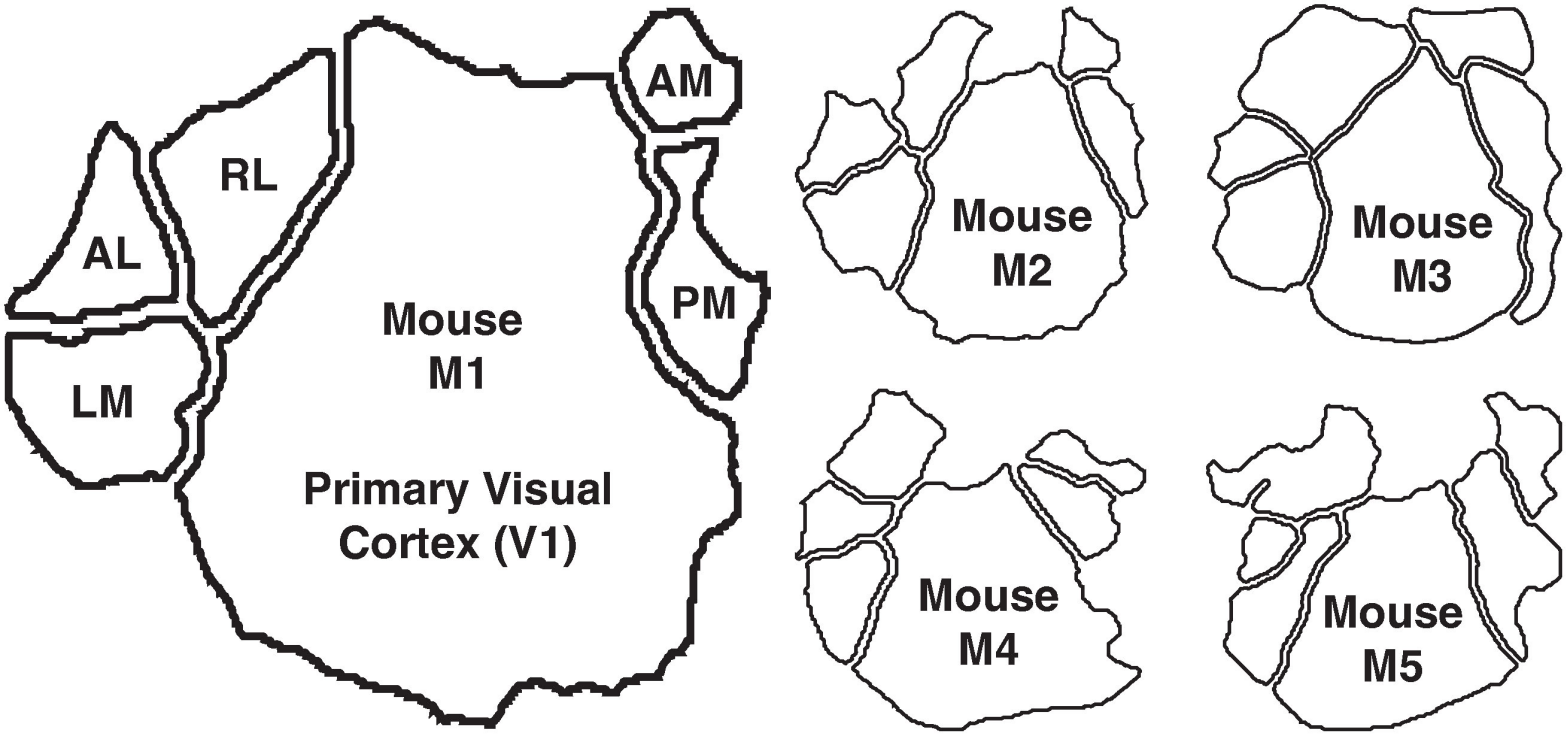
The murine visual cortex, consisting of V1, LM, AL, RL, AM, and PM. Mouse M1 is shown in the large outline and the other four mice M2–M5 are shown in the smaller outlines.

Two-photon imaging data: We next applied the same mixed-flow framework to publicly available empirical data in the form of two-photon calcium-imaging recordings from five mice (Kumar et al., 2021). The dataset includes neuronal responses from six retinotopically defined visual areas: primary visual cortex (V1), lateromedial (LM), anterolateral (AL), rostrolateral (RL), anteromedial (AM), and posteromedial (PM) (Figure 1).

**FIGURE 2:**
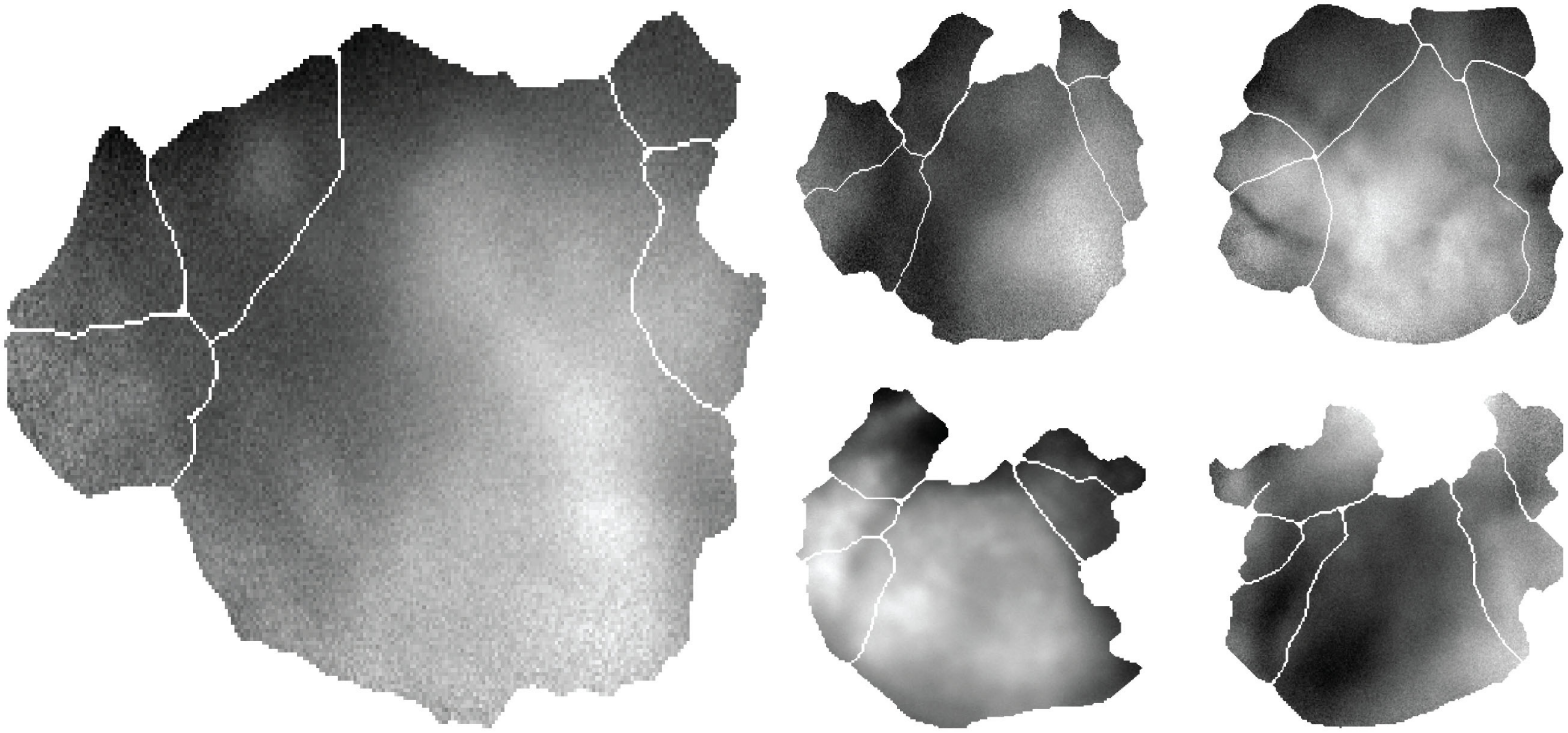
Mice M1-M5 in the same layout as Figure 1, each showing a single frame of fluorescence intensity for the indicator GCaMP6s. We show a segment of these data evolving in time in Supplementary Movie 1.

Visual stimuli consisted of natural movies (30–120 s) and resting-state recordings under a constant grey screen (5 min). ΔF/F traces were pre-processed, aligned to stimulus timing, and grouped by retinotopically defined area (Figure 2).

Model formulation: Although Equation 29 was derived for transformations within a single distribution under changes in observational scale, the same operator can describe transformations *between* marginal distributions of distinct brain regions. For regions *A* and *B* with empirical distributions *p*_*A*_ (*x*) and *p*_*B*_ (*x*), their relationship can be approximated as


$$p_{B}\left(x\right)\approx T_{\alpha ,\beta }\left[p_{A}\left(x\right)\right],$$ (30)

where *T*_α,β_ denotes the mixed entropic–expectation flow operator. This treats inter-regional transformations as the best-fitting reweighting and tilting of *p_A_* in order to recover *p_B_*. The fitted coefficients α and β therefore quantify the relative influence of entropy- and expectation-driven transformations linking the two regions.

For time-series data, the same operator yields a predictive mapping from the activity of region *A* to the estimated signal of region *B*:


$$x_{B}^{e\,s\,t}=x_{A}+\alpha \,\left[l\,o\,g\,q_{A}\,\left(x_{A}\right)-\left\langle l\,o\,g\,q_{A}\right\rangle \right]+\beta \,\left[x_{A}-\left\langle x_{A}\right\rangle \right],$$ (31)

where *q*_*A*_ (*x*_*A*_) is the empirical probability density of *x_A_*. The term with coefficient α reflects local log-density deviations (entropic component) and the term with coefficient β captures global mean deviations (expectation component).

Density estimation and parameter fitting: Empirical densities *q*_*A*_ (*x*_*A*_) were recovered using Gaussian kernel density estimation (KDE) via MATLAB’s *ksdensity* function, which implements Silverman’s rule for bandwidth selection. This produces smooth, data-adaptive estimates suitable for evaluating log-density terms.

For each ordered region pair *A*→*B*, we optimized α and β by minimizing the *L*^2^ prediction error between the model-generated and observed time-series signals using MATLAB’s *fmincon* with the interior-point algorithm. The reverse direction *B*→*A* was modeled separately, allowing directional asymmetries to emerge from independently fitted parameters.

Validation and significance testing: To evaluate generalization, we implemented two complementary validation procedures:

1. A hold-out test: α and β were fitted on the first 80 % of each regional time series and tested on the remaining 20%.
2. A randomized cross-validation: 20 independent 80/20 splits were used to assess robustness to data segmentation. For each split, the coefficient of determination (*R*^2^) was computed between the predicted and empirical target signals. Across all region pairs, the difference between training and test performance was small [Δ*R*^2^ = (3.1 ± 0.4) × 10^−2^] indicating that the model generalizes well and does not overfit.

Statistical significance was assessed via temporal permutation. Each input time series was circularly shifted 1,000 times with random offsets within each session, and the transformation was refitted for each surrogate. *p*-values were computed as the proportion of surrogate *R*^2^ values greater than or equal to the empirical result. Multiple comparisons across all off-diagonal region pairs and mice were corrected using the Benjamini–Hochberg procedure (Benjamini and Hochberg, 1995) (*q* = 0.01), and results were additionally verified using Bonferroni adjustment.

## Results

All results can be reproduced with the accompanying code (see Code Availability).

**FIGURE 3:**
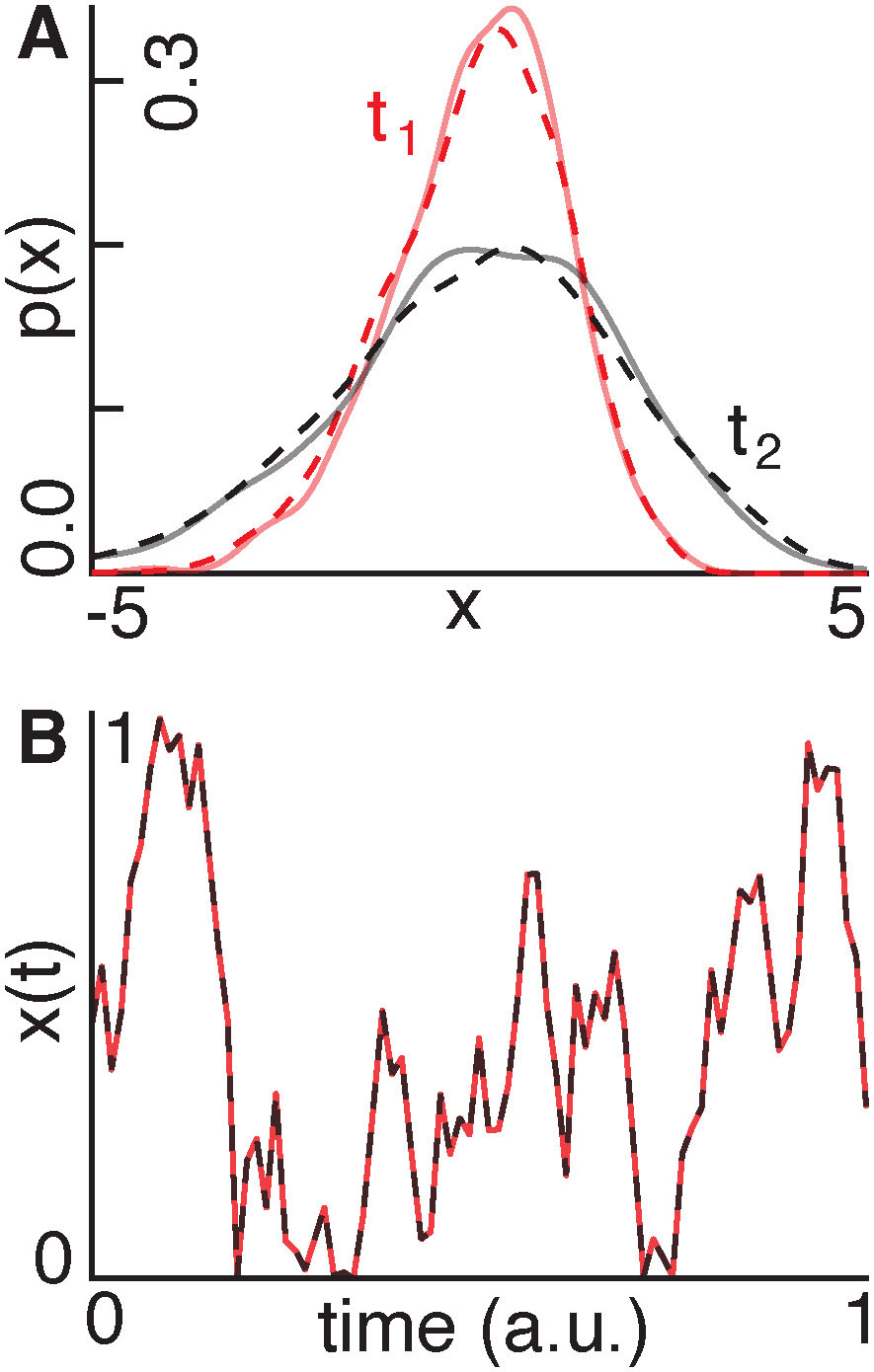
**(A)** We show a Gaussian distribution evolving according to a diffusion process at an early point in time t_1_ (red) and at a later point in time t_2_ (black). The solid and dashed lines indicate the distributions generated using ground-truth and recovered parameters, respectively. **(B)** A signal x(t) evolves according to a stochastic Langevin process with an oscillatory drift using ground-truth (red) and recovered (black) parameters.

Synthetic data: Using known entropy (α) and expectation (β) flow parameters from Equation 29, we created the following two forward-generative models:

1. A Gaussian process undergoing noise-driven diffusion (Figure 3A).
2. A stochastic Langevin process with a sinusoidal drift (Figure 3B).

In the case of the Gaussian process (Figure 3A), the model recovered α and β with errors of 24.4% and 19.8%, respectively. The recovered distributions accurately matched the ground-truth distributions across time, with an average squared Wasserstein-2 distance of 9.3 × 10^−4^, a total variation distance of 0.03, and a mean *L*^2^ error of 9.0 × 10^−4^. In the case of the Langevin process (Figure 3B), the recovered α and β-values deviated from the ground-truth values by 7.1% and 3.0%, respectively. The recovered signal closely tracked the ground-truth trajectory, with a total variation distance of 0.02 and an *L*^2^ error of 0.03.

**FIGURE 4:**
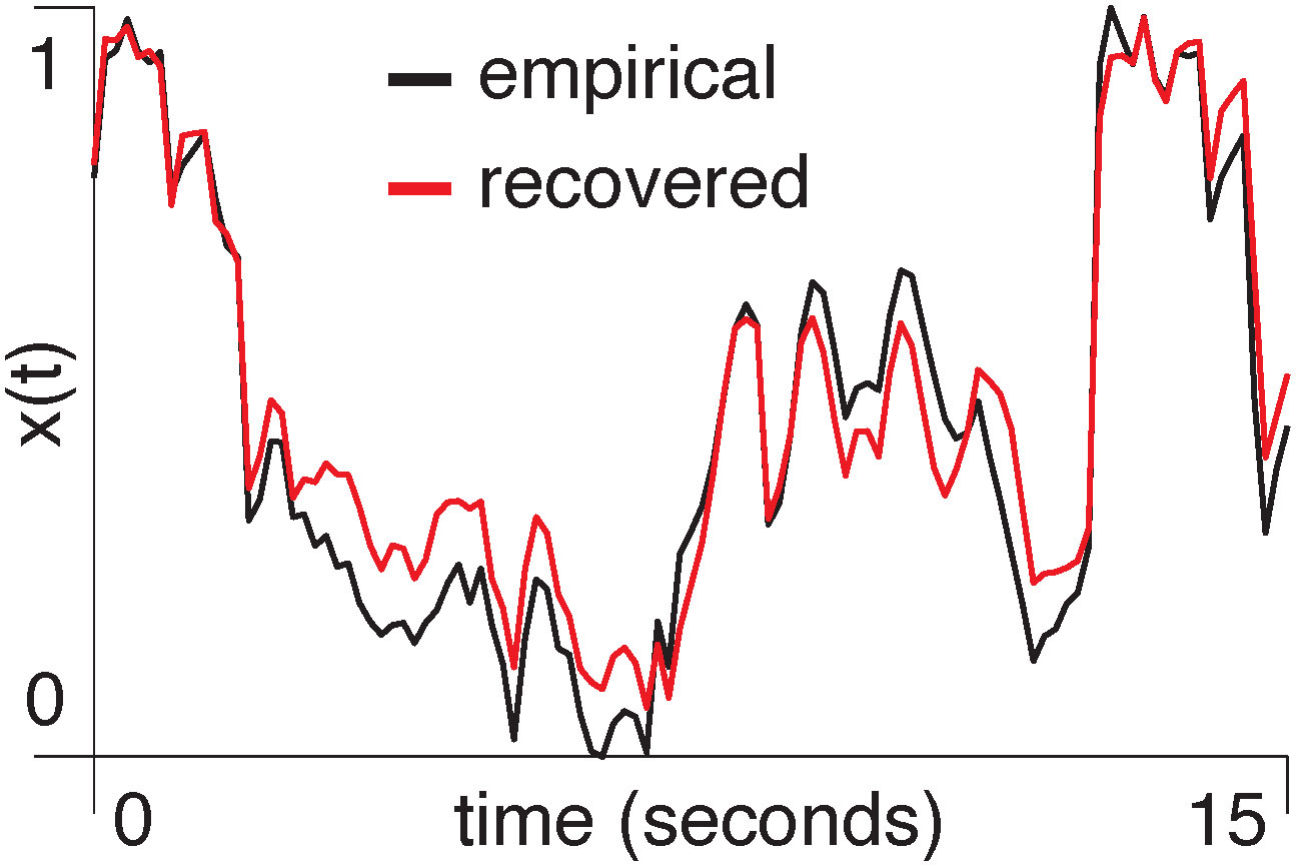
A segment of the normalized first principal component of two-photon signal amplitude from area AM in mouse M3 is shown in black. The red trace shows the result of using V1 to predict activity in AM with the mixed-flow transformation model.

Empirical data: We computed the first principal component of pixel activity within each region of the visual cortex and used the mixed-flow transformation from Equation 29 to model signals within one region, based on another region’s activity. We show an example of using the primary visual cortex (V1) to estimate the anteromedial area (AM) (*R*^2^ = 0.90, *p* = 0.001, Figure 4).

**FIGURE 5:**
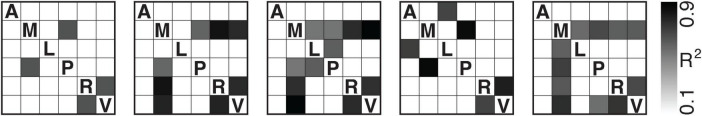
Pairwise directional predictability between brain regions: anterolateral (A), anteromedial (M), lateromedial (L), posteromedial (P), rostrolateral (R), and primary visual cortex (V). Each matrix corresponds to one mouse (M1–M5, left to right). Greyscale values indicate the coefficient of determination (R^2^) for directional prediction strength between each pair of cortical regions. Note that all values remain significant following FDR correction. We show the equivalent results using Bonferroni correction in Supplementary Figure 1.

Correcting for multiple comparisons using false discovery rate (FDR), we performed this same analysis for every pair of regions across mice (Figure 5).

The highest *R*^2^ values which are consistent across all five mice occur between the rostrolateral area and primary visual cortex. Across all region pairs, α averaged (−8.9 ± 8.0) × 10^−6^ and β −1.0 ± 0.1 (see Supplementary Table 1), indicating that expectation-driven transformations dominated the mappings.

## Discussion

In this study, we formalize the link between the geometric structure of probability distributions and their information-theoretic content. Specifically, we show that transformations between zero-mean Gaussian distributions can be decomposed into orthogonal entropic and expectation-based components. The centered symmetric distribution assumption used here serves only as an analytically tractable illustration of orthogonal entropy and expectation flows, not as a biological constraint. We validated this framework on synthetic data and then applied it to two-photon neuroimaging from the murine visual cortex, demonstrating how information geometry can reveal structured transformations between populations.

Our analysis revealed a robust bi-directional transformation between the rostrolateral area (RL) and the primary visual cortex (V1). RL integrates visual input from V1 with movement- and task-related signals (Rasmussen et al., 2021), playing a role analogous to the parietal cortex in primates (D’Souza et al., 2022). The observed reciprocity between RL and V1 therefore suggests a loop consistent with predictive-coding theories, in which visual processing arises from reciprocal exchanges between hierarchical regions (Huang and Rao, 2011; Jurjut et al., 2017; Wang and Burkhalter, 2007).

The link between neural dynamics and information processing shown here also aligns with the efficient coding hypothesis, which posits that neural systems adapt their responses to match the statistical structure of sensory input (Manookin and Rieke, 2023). In our framework, entropic and expectation flows capture this adaptation by adjusting the spread and mean of neural activity. In communication-through-coherence (CTC) models (Fries, 2015), information exchange is most effective when inputs arrive during times of high excitability. Analogously, unpredictable sensory input corresponds to dominant entropic flow that broadens response range, whereas predictable or task-driven states correspond to dominant expectation flow that centers activity on relevant signal averages.

In our formulation, the entropy term quantifies the spread of activity distributions within each region, reflecting intrinsic variability, whereas the expectation term quantifies systematic mean shifts reflecting signal transfer between regions. The fitted coefficients α and β thus separate transformation components driven by shared fluctuations versus structured shifts. Regions with strong shared components exhibit higher joint predictability and lower divergence, whereas those dominated by independent fluctuations exhibit higher entropy but weaker coupling.

Traditional approaches such as Granger causality (Ding et al., 2006) or mutual information (Quian Quiroga and Panzeri, 2009) quantify statistical dependencies between regions but do not specify the form of the transformation linking them. Our framework addresses this gap by modeling how one region’s probability distribution is geometrically transformed into that of another. Under zero-mean Gaussian assumptions, the orthogonality of entropic and expectation flows ensures that these transformation components can be interpreted independently. In summary, we introduce a framework that decomposes information-geometric transformations between neural probability distributions into interpretable information-theoretic flow components. Although demonstrated here in the murine visual cortex, the same approach provides a versatile tool for testing theories of neural function across species, modalities, and scales.

## Data Availability

Publicly available datasets were analyzed in this study. This data can be found here: https://figshare.com/articles/dataset/Data_from_Functional_Parcellation_of_Mouse_Visual_Cortex_Using_Statistical_Techniques_Reveals_Response-Dependent_Clustering_of_Cortical_Processing_Areas_/13476522/1. All MATLAB code used to produce results is made available at: https://github.com/allavailablepubliccode/info_flow.
