# Supplementary figures and images for: Information-theoretic gradient flows in mouse visual cortex
Source: Front Neuroinform. 2025 Oct 30;19:1700481. doi: 10.3389/fninf.2025.1700481 (PMC12611820; doi:10.3389/fninf.2025.1700481)

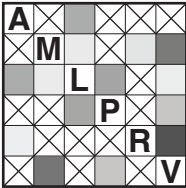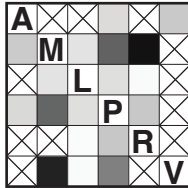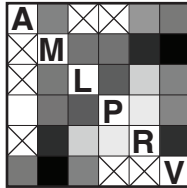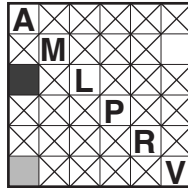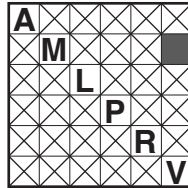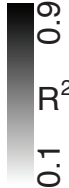

Supplement: Supplementary file 3 [file Image_1.pdf]
